# Research on Quenching of 65Mn Friction Plates in Internal-Circulation Water Channel Molds Based on Finite Element Simulation

**DOI:** 10.3390/ma19112395

**Published:** 2026-06-04

**Authors:** Yu Wang, Ziheng Zhao, Jingang Liu, Xiaoxuan Tu, Gaifen Lu, Jianwen Chen, Ke Liu

**Affiliations:** 1 School of Mechanical Engineering and Mechanics, Xiangtan University, Xiangtan 411105, China; 202231540215@smail.xtu.edu.cn (Y.W.); tuxiaoxuan@xtu.edu.cn (X.T.); lgf9557@sina.com (G.L.); 2 JiangLu Machinery Electronics Group Co., Ltd., Xiangtan 411105, China; wyuone@163.com (J.C.); liuke820x@126.com (K.L.)

**Keywords:** friction plate, hardness, mold, pressure quenching simulation, 65Mn steel, hardness uniformity, warpage deformation

## Abstract

To address uneven surface hardness distribution in 65Mn external tooth friction plates after furnace quenching and disc mold tempering, we adopted an integrated quenching and forming process, using an internal-circulation mold. By simultaneously implementing pressure forming and quenching within the internal-circulation mold, the hardness uniformity of the friction plate during forming was improved, effectively suppressing warping deformation. A multi-field coupled model of the friction plate quenching in the internal-circulation mold was established to simulate the dynamic evolution of the temperature field, the microstructural transformation, and the stress field, thus obtaining the complete heat treatment response of the martensitic transformation. The experimentally observed microstructure agreed well with the simulation results. Data analysis showed that after quenching in the internal-circulation mold, the surface hardness difference of a single friction plate was reduced from 3 HRC to 0.9 HRC, and the end face runout decreased from 0.1–0.15 mm to no more than 0.06 mm, significantly improving the product’s dimensional accuracy and performance consistency.

## 1. Introduction

Integrated transmission devices widely adopt a wet-type shift clutch, with oil as the cooling medium. The input end is connected to the engine, and the output end is connected to the driving wheels. Because the oil pressure is controlled during engagement, the driving and driven friction plates are combined, and power is transmitted through the frictional force between the plates, affecting the power performance and handling of the entire vehicle [1,2]. Currently, the commonly used process scheme for friction plates is: cutting (cold-rolled sheet) → precision punching → atmosphere furnace quenching + pre-hardening + die-casting disc-shaped tempering → surface treatment [3,4,5]. However, the atmosphere furnace quenching process for friction plates is prone to cause severe warping deformation. The actual size of a single-disc-shaped workpiece may sometimes exceed the design tolerance, resulting in a low qualification rate for the single-disc-shaped workpiece. Moreover, due to the use of a multi-layer horizontal loading method for the friction plates, cooling efficiency also varies significantly between workpieces and between different regions of a single plate, thereby affecting some workpieces located in areas with a lower cooling rate (i.e., workpieces located in the middle of a multi-layer horizontal loading). During quenching, the martensitic transformation is not sufficient for some workpieces, resulting in uneven surface hardness distribution in the single friction plate [6,7,8]. Therefore, the industrial production of friction plates requires systematic experimental research and strict parameter control.

To address uneven surface hardness distribution caused by non-uniform martensitic transformation in a 65Mn [9,10,11] external tooth friction plate, this study proposes a pressure-quenching integrated process for a 65Mn external tooth friction plate. This process combines pressure-quenching treatment with an internal-circulation water channel mold during the quenching stage, and the process is analyzed by finite element simulation [12,13,14,15,16,17,18]. This approach can more intuitively reflect changes in temperature, microstructure and pressure in the friction plate during the quenching stage when using an internal-circulation water channel mold for pressure quenching. Then, by analyzing the content of the martensite microstructure, the hardness distribution can be reflected [19,20,21,22]. Compared with the traditional atmosphere furnace quenching process, application of a pressure-quenching integrated process in internal-circulation water channel molds can relatively reduce uneven surface hardness distribution and deformation.

## 2. Materials and Methods

### 2.1. Overview of Simulation

**Figure 1:**
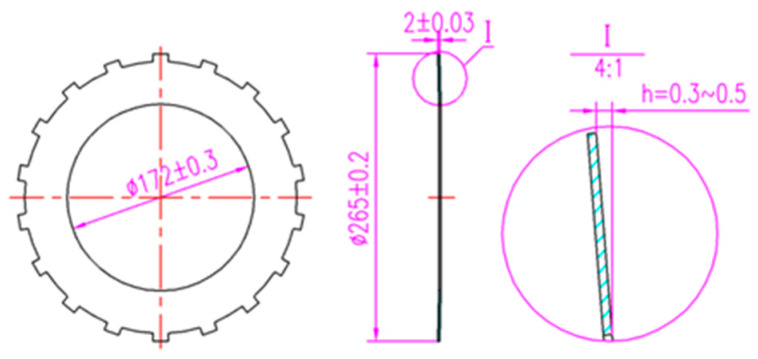
External tooth friction plate.

The structural dimensions of a typical wet-type shift clutch external tooth friction plate are shown in Figure 1. The plate has a thickness of 2 mm, is made of 65Mn steel with a hardness of 28–35 HRC, and requires a warpage control range of 0.3–0.5 mm. When 65Mn external tooth friction plates are quenched in an atmosphere furnace, severe warping deformation tends to occur. The qualification rate for single-disc-shaped workpieces is only about 85%, and their runout reaches 0.1–0.15 mm. Furthermore, the surface hardness variation among friction plates produced in the same batch is 5 HRC, while that within a single workpiece is 3 HRC.

**Figure 2:**
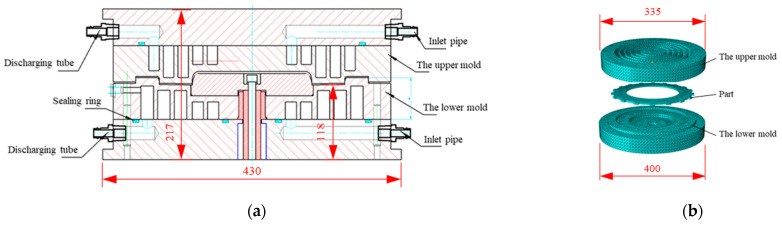
Finite element model of water-cooled mold quenching: (**a**) Two-dimensional schematic diagram; (**b**) three-dimensional schematic diagram.

When the 65Mn friction plates were placed in the dual-station multi-frequency induction heat treatment equipment—ZTVPYC-2S (Shiyan High Frequency Science and Technology Industry & Trade Co., Ltd., Hubei, China) for the internal circulating water channel mold, these plates were heated to 850°C through induction heating. This quenching temperature is slightly higher than the 850 °C used in atmosphere furnaces. The plates were then placed into the internal-circulation water channel mold and quenched with cooling water at 25 °C, finally cooling to room temperature (25 °C). The finite element mesh division of the model is shown in Figure 2.

### 2.2. Mathematical Model

#### 2.2.1. Temperature Field

The temperature field satisfies the transient nonlinear heat conduction equation [23]. The mathematical model of the temperature field is as follows:

(1) $$\frac{\partial }{\partial x}\left(\lambda \frac{\partial T}{\partial x}\right)\;+\;\frac{\partial }{\partial y}\left(\lambda \frac{\partial T}{\partial y}\right)\;+\;\frac{\partial }{\partial z}\left(\lambda \frac{\partial T}{\partial z}\right)\;+\;Q\;=\;\rho c\frac{\partial T}{\partial t},$$

Initial conditions:

(2) $${\left.T\right|}_{t\;=\;0}\;=\;T_{0}$$

Boundary conditions:

(3) $$\begin{array}{ll} {\left.-\lambda \frac{\partial T}{\partial n}\right|}_{s} & \;=\;H_{k}\left(T_{w}-T_{c}\right)\;+\;\sigma \varepsilon \left(T_{w}^{4}-T_{c}^{4}\right)\\ & \;=\;H_{k}\left(T_{w}-T_{c}\right)\;+\;H_{s}\left(T_{w}-T_{c}\right)\\ & \;=\;H\left(T_{w}-T_{c}\right) \end{array}$$

In the formula: T represents temperature; λ is the thermal conductivity coefficient; ρ is the density; c is the specific heat capacity; t is time; Q is the latent heat of phase transformation; H is the overall heat transfer coefficient; $H_{k}$ is the convective heat transfer coefficient; $H_{s}$ is the radiative heat transfer coefficient; $T_{w}$ is the known surface temperature of the workpiece; $T_{c}$ is ambient temperature; During the quenching process, T changes with t. λ, c, and Q  are all functions of T. When the latent heat of phase transformation and the plastic work generated heat are neglected, Q = 0.

#### 2.2.2. Microstructure Field

The calculation models for the phase transformation models of 65Mn steel are generally divided into two types, namely, diffusion type and shear type. Martensitic transformation is recognized as a shear-type phase transformation, the martensitic phase transformation belongs to the shear-type phase transformation, and its phase transformation kinetics conform to the Koistinen–Marburger (K–M) equation [24]:

(4) $$f\;=\;f_{\gamma }\left\{1-exp\left[-\alpha \left(M_{s}-T\right)\right]\right\},$$

In the formula, $f_{\gamma }$ represents the volume fraction of austenite before the martensitic phase transformation begins; *α* is the phase transformation kinetic parameter; $M_{s}$ is the starting temperature of the martensitic transformation; *T* is the instantaneous temperature.

#### 2.2.3. Stress and Strain Field

The strains that occur during the quenching process of 65Mn steel mainly include elastic strain, plastic strain, thermal strain, phase transformation strain and transformation plasticity strain. The total strain increment is related as follows:

(5) $$d\varepsilon \;=\;d\varepsilon^{e}\;+\;d\varepsilon^{p}\;+\;d\varepsilon^{th}\;+\;d\varepsilon^{tr}\;+\;d\varepsilon^{tp},$$

In the formula, dε represents the total strain increment, $d\varepsilon^{e}$ represents the elastic strain increment, $d\varepsilon^{p}$ represents the plastic strain increment, $d\varepsilon^{th}$ represents the thermal strain increment, $d\varepsilon^{tr}$ represents the phase transformation strain increment, and $d\varepsilon^{tp}$ represents the transformation plasticity strain increment.

Thermal strain is the strain generated within the workpiece due to the temperature gradient inside it. Phase transformation strain is the additional strain resulting from the difference in specific volume between the parent and product phases. Transformation plasticity strain is the plastic strain generated during the phase transformation process even when the internal stress has not reached the yield limit. Thermal strain, phase transformation strain, and transformation plasticity strain can be calculated respectively using the following formulas:

(6) $$d\varepsilon^{th}\;=\;\alpha dT,$$

(7) $$d\varepsilon^{tr}\;=\;\sum_{i\;=\;1}^{n}\beta_{T}^{i}df_{i},$$

(8) $$d\varepsilon^{tp}\;=\;3K\left(1-f_{i}\right)S_{ij}df_{i},$$

In the equation, α represents the thermal expansion coefficient, $\beta_{T}^{i}$ is the expansion coefficient of the new phase when it undergoes transformation at temperature T, $df_{i}$ is the increment of the new phase, $S_{ij}$ is the stress component tensor, and K is the phase transformation plasticity coefficient. After obtaining the strain variation within the workpiece, the stress field of the workpiece can be obtained by calculating the thermo-elasto-plastic stress–strain constitutive equation.

#### 2.2.4. Heat Transfer Coefficient

Radiation heat transfer is neglected in the quenching process of 65Mn steel, and is simplified to the convective heat transfer between the workpiece (including the mold) and the quenching medium, as well as the internal heat conduction of the workpiece itself. The convective heat transfer coefficient [25] is affected by many factors and can be expressed by the following functional relationship:

(9) $$h_{c}\;=\;f\left(u,T_{w},T_{f},\lambda ,c,\rho ,\mu ,L,\ldots \right),$$

In the formula: u represents the fluid flow velocity; λ is the thermal conductivity coefficient; ρ is the density; c is the specific heat capacity; $T_{w}$ is the surface temperature of the sample; $T_{f}$ is the temperature of the surrounding fluid; μ is the dynamic viscosity; L is the characteristic geometric dimension of the workpiece. Additionally, the convective heat transfer coefficient is also related to the flow state of the liquid. Generally, its value is generally lower when the liquid is in laminar flow rather than in turbulent flow.

### 2.3. Material Composition

Because the content of each element in 65Mn steel varies within a certain range, to simplify the calculation, the measured composition of a certain batch of semi-finished products using the spark 8000 direct reading type spectrometer (NAKAR Testing Technology Co., Ltd., Beijing, China) was adopted as the calculation basis. For details, please refer to Table 1.

### 2.4. Thermal Physical Parameters

**Figure 3:**
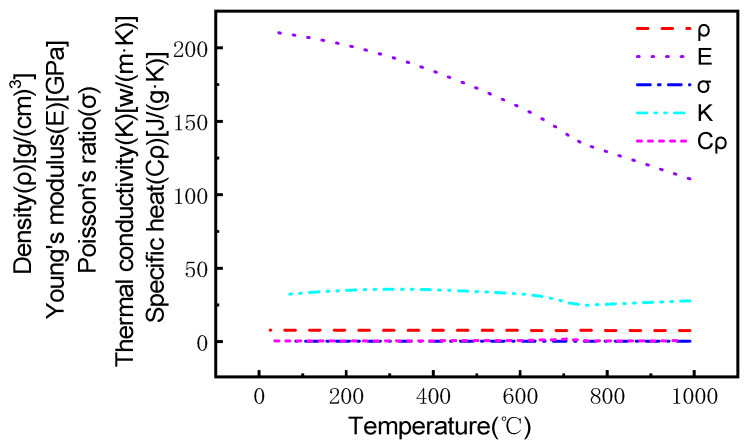
Thermal physical properties of 65Mn steel.

During the quenching process, the specific heat capacity, thermal conductivity, elastic modulus, thermal expansion coefficient, and surface comprehensive heat transfer coefficient of the material will all change with the variation of temperature. These thermophysical parameters are obtained by calculating the chemical composition and elemental content of 65Mn steel. The calculation results of the specific heat capacity, thermal conductivity, elastic modulus, Poisson’s ratio, and linear thermal expansion coefficient of 65Mn steel are shown in Figure 3.

### 2.5. Heat Transfer Coefficient and C Curve

**Figure 4:**
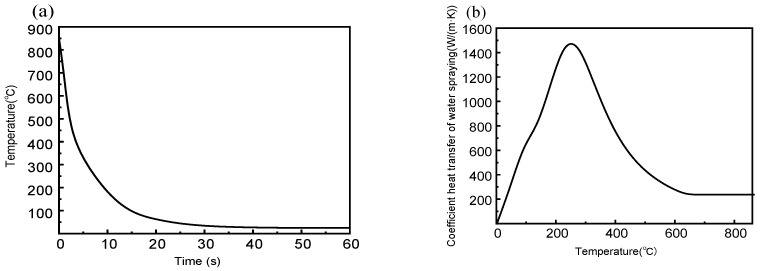
Cooling curve of the workpiece, and the heat transfer coefficient of the water. (**a**) The measured cooling curve; (**b**) the heat transfer coefficient of the water.

**Figure 5:**
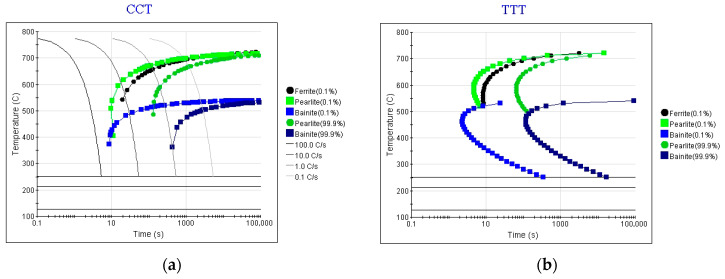
TTT and CCT curves for 65Mn steel. (**a**) TTT curve; (**b**) CCT curve.

During the cooling process of the friction plate, multiple heat transfer modes are involved simultaneously. Therefore, separate settings are required for convective heat exchange between the sheet material and the air, contact heat transfer between the sheet material and the mold, convective heat exchange between the mold and the cooling water, and thermal radiation (emissivity and Stefan–Boltzmann constant) of the sheet material. To determine the heat transfer coefficient of the water medium, the corresponding cooling curve is measured experimentally, and then the heat transfer coefficient is calculated through inverse heat transfer (as shown in Figure 4). Similarly, the TTT and CCT curves shown in Figure 5 are calculated based on the elemental content of 65Mn steel.

The specific scheme for measuring the cooling curve was as follows: A blind hole with a diameter of 1 mm was drilled on the workpiece surface. A thermocouple was fixed in the blind hole processed on the workpiece, and the thermocouple was connected to the temperature acquisition system. Next, the workpiece was placed in the heating furnace and heated to 850 °C for 60 min to ensure that the temperature at all positions was uniform. The workpiece was then placed quickly in the internal-circulation water channel mold, and quenching was performed under the condition of a water temperature of 25 °C. The thermocouple transferred the temperature change values of the near surface of the workpiece to the temperature acquisition module during the cooling process, and the data was read and saved.

## 3. Results

### 3.1. Simulation Results

**Figure 6:**
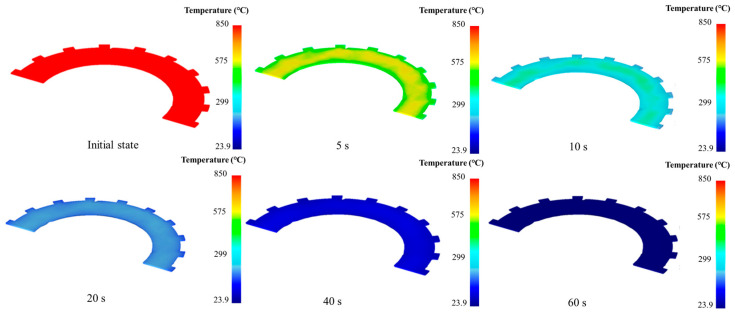
Temperature field evolution of the friction plate during quenching.

Figure 6 shows the temperature field changes of the friction plates at different times during the quenching process. It can be seen that the overall quenching is relatively uniform and the cooling effect is good. By 40 to 60 s, the plates have basically cooled to room temperature.

**Figure 7:**
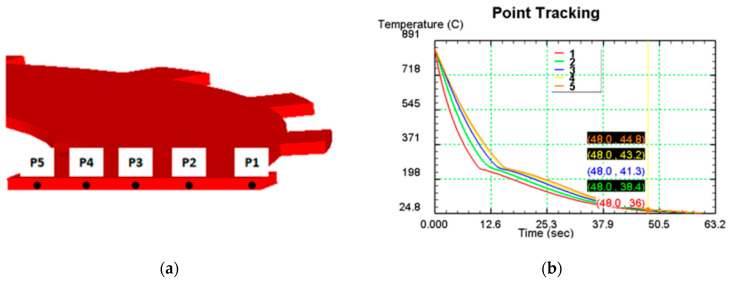
Curve showing the temperature changes of each node of the friction plate over time: (**a**) Node position map; (**b**) temperature curve.

Five key nodes were evenly selected on the cross-section of the friction plate, and their temperature variation curves over time are shown in Figure 7. At 48 s, the temperatures of P1, P2, P3, P4, and P5 were 36 °C, 38.4 °C, 41.3 °C, 43.2 °C, and 44.8 °C, respectively. Node P1 was slightly closer to the tooth and the outer edge than other nodes, leading to a marginally higher cooling rate. However, because the thickness of the friction plate was relatively thin (2 mm), the temperature difference was negligible, and was only 8.8 °C compared to the temperature maximum node P5. Moreover, all nodes reached room temperature after around 50 s. Please refer to Figure 7 for details. This indicates that the temperature distribution of the friction plate is mainly related to the 60Mn material and the quenching medium during the quenching process. It can be seen in the figure that the temperatures at points P1, P2, P3, P4, and P5 all decrease with time. This transition occurs at approximately 12 s, at a temperature of about 270 °C. This suggests that during the pressure quenching process in the internal-circulation mold, the heat exchange is more intense; it also proves that the heat transfer coefficient reaches its maximum value at approximately 270 °C, which is consistent with the description in Figure 4 mentioned above.

**Figure 8:**
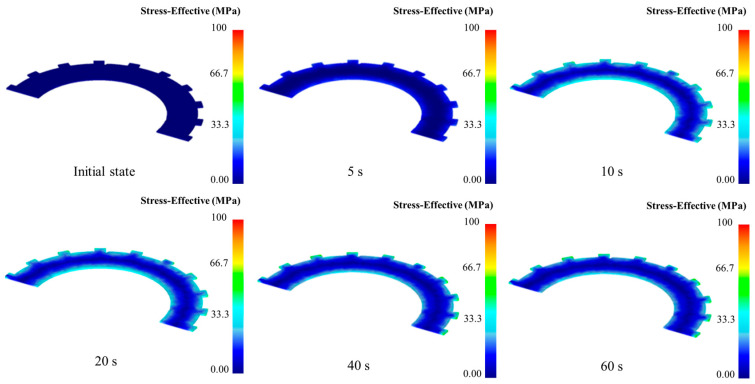
Effective stress field evolution of the friction plate during quenching.

Figure 8 shows the stress field conditions of the friction plate during the quenching process at different times. It can be seen that throughout the quenching process, the maximum stress occurs on the outer edge of the tooth near the beginning of cooling, around 10 to 20 s. Subsequently, as the quenching progresses, the stress gradually increases. And when the quenching cooling is completed, the outer edge of the tooth is still the maximum, at around 40 MPa.

**Figure 9:**
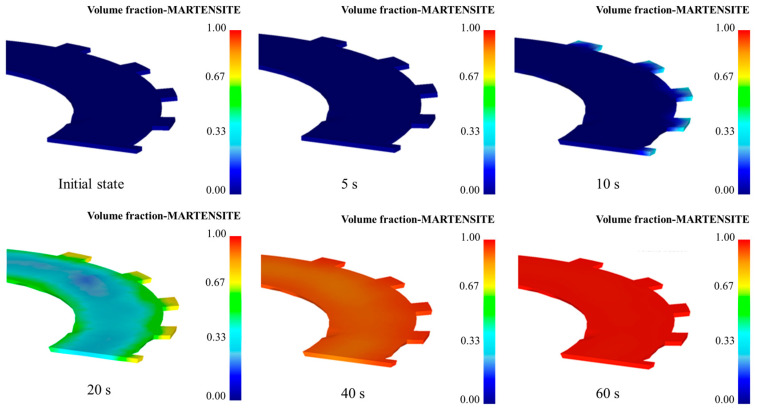
Martensite volume fraction evolution of the friction plate during quenching.

Figure 9 shows the microstructural transformation of the friction plate during the quenching process at different times. It can be seen that at 10 s, the friction plate initially undergoes an austenite-to-martensitic transformation starting from the outer edge of the tooth section. At 60 s, the entire workpiece has completed the martensitic transformation, and the original martensite volume of 0% has changed to 100%.

The friction plate undergoes a complete transformation to the martensite microstructure when quenched and cooled in the internal-circulation water channel mold for 60 s. In addition, hardness is strongly correlated with the content of the material’s microstructure, so the surface hardness of the friction plate is also distributed uniformly like the microstructure.

### 3.2. Experimental Results

After 60 friction plates were quenched in the internal-circulation water channel mold, they were neatly placed into the disc-shaped tempering mold, and the clamping fixture was then locked. The disc-shaped tempering treatment was carried out at a temperature of 550 °C for 120~150 min according to the procedure. The plates were cooled by forced-air cooling, and five plates were randomly selected as pieces to test the hardness and disc-shaped dimensions. The results are shown in Table 2. From the results in Table 2, it can be seen that the hardness of the five tested plates ranges from 33.7 to 34.9 HRC, the warpage range is (0.36~0.45) mm, the single-piece hardness dispersion is ≤0.9 HRC, and the total runout value is ≤0.06 mm.

Compared with the atmosphere furnace quenching and disc mold tempering process for the friction plates, not only did the maximum surface hardness dispersion of each single piece decrease from 3 HRC to 0.9 HRC, but the total runout of the single external tooth friction plate also decreased, from 0.1~0.15 mm to no more than 0.06 mm.

**Figure 10:**
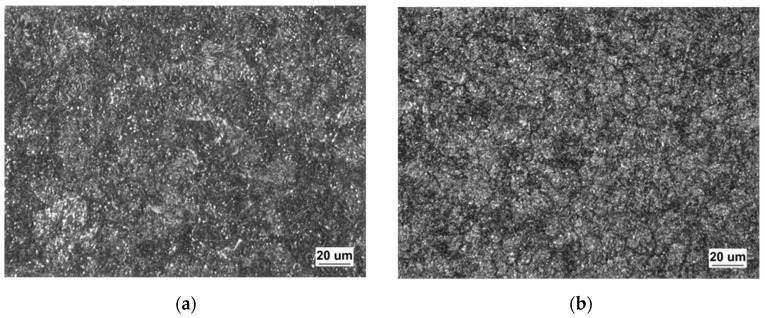
Microstructure: (**a**) Quenched structure; (**b**) tempered structure.

In addition, the microstructure of the friction plates that had undergone quenching but not subsequent tempering treatment was analyzed using a 4XC-II three-lens metallographic microscope (Shanghai Optical Instrument Factory, Shanghai, China). Predominantly, the as-quenched microstructure was martensite (Figure 10a), a finding which indirectly verified the accuracy of the simulation. After tempering, the microstructure transformed into sorbite (Figure 10b). The experiment shows that in internal-circulation water channel molds, the material transforms into a fully martensitic structure, reducing the range of surface hardness distribution. Then, after tempering, the martensitic structure transforms into sorbite.

## 4. Discussion

This study combines finite element multi-field coupling simulation with experimental validation to systematically analyze temperature, stress, and microstructural evolution in 65Mn external tooth friction plates during pressure quenching in an internal-circulation water channel mold. The results show that the friction plates achieve the martensitic transformation within 60 s, with uniform surface hardness, single-piece hardness deviation ≤0.9 HRC, and total runout ≤0.06 mm, representing a significant improvement compared to conventional atmosphere furnace quenching and die-cast disc tempering processes. It may be observed in Figure 6 and Figure 7 that the cooling rates at five key nodes are consistent, indicating that the thin-wall structure favors uniform temperature distribution. Figure 8 shows stress concentration at the tooth edge, but the overall stress remains below the material yield strength, effectively suppressing warpage and microcrack formation. The microstructural evolution shown in Figure 9 and Figure 10 and the hardness results in Table 2 further confirm the accuracy of the simulation predictions and the reliability of the process. From the perspective of prior research and process assumptions, the coupled effect of pressure constraint and circulating water cooling not only optimizes thermal stress distribution but also promotes microstructural uniformity, thereby significantly enhancing mechanical performance and dimensional stability. During the quenching process, the 65Mn external tooth friction plate was changed from being quenched in the original atmosphere furnace to being quenched in internal-circulation water channel molds. This change effectively controlled the surface hardness deviation and deformation amount. In a broader context, this study provides theoretical and practical guidance for the industrial production of high-precision friction components and offers a reference model for simulating and optimizing the structure–performance relationships in similar steel heat treatment processes. Overall, the results demonstrate that reasonable design of quenching process parameters and mold structure can ensure microstructural uniformity while improving workpiece performance consistency, thereby enhancing the reliability and durability of transmission system friction plates.

## 5. Conclusions

Based on the above discussion, a multi-field coupling simulation model of 65Mn external tooth friction plates during pressure quenching in an internal-circulation water channel mold was established and validated experimentally. The study demonstrates that the friction plates completed the martensitic transformation within 60 s, achieving uniform surface hardness, single-piece hardness deviation ≤0.9 HRC, and total runout ≤0.06 mm, which is significantly superior to conventional atmosphere furnace quenching and die-cast disc tempering processes. This confirms the effectiveness of the synergistic combination of pressure constraint and circulating water cooling in optimizing thermal stress distribution and promoting microstructural uniformity, directly improving dimensional stability and performance consistency. Furthermore, the multi-field coupling simulation approach provides a reference model for the structure–performance relationships of similar thin-walled steel components, offering theoretical guidance and practical support for process parameter optimization, mold design improvement, and industrial production. Overall, this study not only achieves a dual optimization of hardness and geometric accuracy of the friction plates, but also provides solid technical support for the reliability and durability of high-precision transmission components in practical applications.

1. A multi-field coupling model for the quenching of 65Mn external tooth friction plates in an internal-circulation water channel mold was established to simulate changes in temperature, microstructure and pressure during the quenching process of 65Mn external tooth friction plates in an internal-circulation water channel mold.

2. By simulating the microstructural transformation of 65Mn external tooth friction plates, it was found that the microstructure could be transformed into martensite after 60 s of water cooling, and the hardness distribution of the friction plate after quenching in the internal-circulation water channel mold was uniform.

3. It was found that when 65Mn external tooth friction plates were quenched in the internal-circulation water channel mold, the total runout of a single external tooth friction plate could be reduced from 0.1 to 0.15 mm to no more than 0.06 mm, and the maximum hardness dispersion of the single surface of the friction plate was reduced from 3 HRC to 0.9 HRC.

## Figures and Tables

**Table 1:** Chemical composition (wt.%) of the 65Mn steel used in the calculation.

65Mn	C	Si	Mn	P	S	Fe
Component range (wt.%)	0.62~0.70	0.17~0.37	0.90~1.20	≤0.035	≤0.035	Balance
Calculation value	0.64	0.22	0.99	0.022	0.02	Balance

**Table 2:** Hardness and disc shape test results.

Specimen No.	Rockwell Hardness Hardness Dispersion/HRC			Range Hardness Dispersion/HRC	Warpage/mm	Total Runout/mm
1#	34.3	34.6	34.6	0.3	0.39~0.44	0.05
2#	34.5	34.3	34.4	0.2	0.38~0.42	0.04
3#	34.6	34.9	34.5	0.4	0.39~0.42	0.03
4#	33.7	33.7	34.6	0.9	0.38~0.41	0.03
5#	34.5	34.4	34.5	0.1	0.38~0.42	0.04

## Data Availability

The original contributions presented in this study are included in the article. Further inquiries can be directed to the corresponding authors.
